# Cost analysis of rapid diagnostics for drug-resistant tuberculosis

**DOI:** 10.1186/s12879-018-3013-0

**Published:** 2018-03-02

**Authors:** Erik J. Groessl, Theodore G. Ganiats, Naomi Hillery, Andre Trollip, Roberta L. Jackson, Donald G. Catanzaro, Timothy C. Rodwell, Richard S. Garfein, Camilla Rodrigues, Valeriu Crudu, Thomas C. Victor, Antonino Catanzaro

**Affiliations:** 1 Department of Family Medicine and Public Health, University of California San Diego, 9500 Gilman Dr, #0994, San Diego, CA USA; 2 VA San Diego Healthcare System, San Diego, CA USA; 3 Department of Biomedical Sciences, Stellenbosch University, Cape Town, South Africa; 4 Department of Medicine, University of California, San Diego, CA USA; 5 University of Arkansas, Little Rock, USA; 6 Hinduja National Hospital, Mumbai, India; 7 Microbiology and Morphology Laboratory, Institute of Phthisiopneumology, Chisinau, Moldova

**Keywords:** Drug-resistant tuberculosis, Diagnosis, Cost-effectiveness, Time to result

## Abstract

**Background:**

Growth-based drug susceptibility testing (DST) is the reference standard for diagnosing drug-resistant tuberculosis (TB), but standard time to result (TTR) is typically ≥ 3 weeks. Rapid tests can reduce that TTR to days or hours, but accuracy may be lowered.

In addition to the TTR and test accuracy, the cost of a diagnostic test may affect whether it is adopted in clinical settings. We examine the cost-effectiveness of rapid diagnostics for extremely drug-resistant TB (XDR-TB) in three different high-prevalence settings.

**Methods:**

1128 patients with confirmed TB were enrolled at clinics in Mumbai, India; Chisinau, Moldova; and Port Elizabeth, South Africa. Patient sputum samples underwent DST for first and second line TB drugs using 2 growth-based (MGIT, MODS) and 2 molecular (Pyrosequencing [PSQ], line-probe assays [LPA]) assays. TTR was the primary measure of effectiveness. Sensitivity and specificity were also evaluated. The cost to perform each test at each site was recorded and included test-specific materials, personnel, and equipment costs. Incremental cost-effectiveness ratios were calculated in terms of $/day saved. Sensitivity analyses examine the impact of batch size, equipment, and personnel costs.

**Results:**

Our prior results indicated that the LPA and PSQ returned results in a little over 1 day. Mean cost per sample without equipment or overhead was $23, $28, $33, and $41 for the MODS, MGIT, PSQ, and LPA, respectively. For diagnosing XDR-TB, MODS was the most accurate, followed by PSQ, and LPA. MODS was quicker and less costly than MGIT. PSQ and LPA were considerably faster but cost more than MODS. Batch size and personnel costs were the main drivers of cost variation.

**Conclusions:**

Multiple factors must be weighed when selecting a test for diagnosis of XDR-TB. Rapid tests can greatly improve the time required to diagnose drug-resistant TB, potentially improving treatment success, and preventing the spread of XDR-TB. Faster time to result must be weighed against the potential for reduced accuracy, and increased costs.

**Trial registration:**

ClinicalTrials.gov Identifier: NCT02170441.

**Electronic supplementary material:**

The online version of this article (10.1186/s12879-018-3013-0) contains supplementary material, which is available to authorized users.

## Background

Tuberculosis (TB) remains a leading cause of mortality globally [1]. Drug-resistant strains of TB (DR-TB) have emerged, mostly due to inadequate or incomplete treatment [2]. To diagnose and then appropriately treat DR-TB, it is important to conduct drug susceptibility testing (DST), especially in regions known to have high levels of DR-TB [2, 3]. Effectively diagnosing and treating drug-resistant TB is key to reducing transmission [4], improving treatment outcomes and lowering mortality.

In response to the long time to result (TTR) inherent in conventional reference methods for diagnosing DR-TB, novel, rapid DST methods are becoming available [5–7]. In addition, an international consortium was established to evaluate microbiological and molecular assays for quickly and efficiently detecting DR-TB [8, 9].

In addition to TTR, there are a number of other important characteristics of DR-TB assays that should be considered, including the accuracy and the cost of new tests [10, 11]. A test that is rapid but not accurate may be of questionable value, and an accurate and rapid test may be inaccessible if it is costly [12]. Thus it is important to consider all three of these characteristics (test accuracy, time to result, and cost of the test) when evaluating novel DR-TB diagnostics [13]. The objective of our study was to compare the cost-effectiveness of three currently available, rapid diagnostic tests to MGIT960 DST, a WHO recommended reference standard [1, 14, 15]. The data will allow experts to gauge whether shorter time to result or increased accuracy is worth additional costs for certain new diagnostic tests.

## Methods

### Study design

The parent study design and methods have been previously described elsewhere [9]. Briefly, the goal of the overall study was to compare the TTR and the sensitivity/specificity of three rapid tests for diagnosis of extremely drug resistant tuberculosis (XDR-TB) to MGIT DST. XDR-TB is defined as resistance to both isoniazid and rifampicin, as well as any one of the fluoroquinolones, and any one of the injectable anti-TB drugs [1]. The diagnostic tests were run in parallel on study participants at three large TB clinics located in areas of elevated XDR-TB prevalence. Participants suspected of having XDR-TB were enrolled (see Additional file 1: Table S1 for inclusion/exclusion criteria) [9]. Biological specimens and interview data were collected at baseline and 52-week follow-up visits. In addition, medical record reviews were conducted at baseline, 30 days post-enrollment and 52 weeks post-enrollment. Enrollment occurred between June 2012 and June 2013. Participants were not compensated for participation, but travel costs were reimbursed when patients traveled an hour or more for research-related visits.

### Study sites

The three study sites were located in Mumbai, India; Port Elizabeth, South Africa; and Chisinau, Moldova.

#### India

The P.D. Hinduja National Hospital (PD-HNH) and Medical Research Centre (MRC), is a tertiary care center in central Mumbai, India. The Pulmonary Department at the PD-HNH is the busiest in Mumbai and is the referral center for MDR and XDR-TB cases from the Mumbai and the state of Maharashtra. In a previous study of the patient population at this clinic, 80% of samples obtained were found to be resistant to one or more standard TB medications, while 51% were resistant to more than one drug [16].

#### Moldova

The Phthisiopneumology Institute (PPI) in Chisinau, Moldova is the central unit of the National TB Control Programme. It is a medical consultation, scientific research, and training center that leads all TB patient services across Moldova. Moldova has a high prevalence of drug resistant TB, with 24% of new and 62% of previously treated TB patients having MDR-TB [17].

#### South Africa

According to the WHO, South Africa has a high number of incident TB cases and a high prevalence of drug-resistant TB [9]. At the Port Elizabeth site, patients were enrolled at six primary health care facilities and one regional hospital. The decentralized enrollment resulted in a lower prevalence of drug resistance at this site [1].

##### Experience and training of laboratory personnel

Each of the study sites described above had existing clinical laboratories that had been in operation for many years and adhered to international safety standards. The study required each site to identify a laboratory technician with at least one year experience conducting TB testing. Each site had ongoing experience conducting drug-resistance testing for TB using the MGIT and line-probe assay. For these tests, each site was sent a set of samples to test and site results were compared to previously established findings at the coordinating center (UCSD). The lab technician from each site also jointly attended a week-long training for the MODS at the Universidad Peruana Cayetano Heredia in Lima, Peru and a week-long training on Pyrosequencing with Dr. Grace Lin at the California Department of Public Health, Richmond, California. Training consisted of lecture, lab instruction, and hands on training to establish standardized testing procedures across sites.

### Inclusion/exclusion criteria

To be eligible for the study, participants had to a) be at least 5 years of age; b) have provided informed consent or had ability and willingness of subject or legal guardian/representative to provide informed consent; c) known to be AFB sputum smear-positive (defined as 1+ or greater within prior 14 days), positive on GeneXpert, or present clinically with high suspicion of active TB and:

- Had previously received > 1 month of treatment for a prior TB episode or
- Were failing TB treatment with positive sputum smear or culture after ≥3 months of a standard TB treatment or
- Had had close contact with a known drug-resistant TB case or
- Were newly diagnosed with MDR-TB within the last 30 days or
- Were previously diagnosed with MDR-TB and failed TB treatment with positive sputum smear or culture after ≥3 months of a standard MDR-TB treatment regimen.
- Exclusion criteria were a) institutionalized; b) unable to provide at least 7.5 ml sputum (1st and 2nd samples combined) or c) had results from second line DST performed within the last 3 months.

### Study measures

#### Effectiveness

The TTR of each assay was the primary effectiveness outcome. TTR was defined as “the number of days from initiation of testing to recording of final results of **all seven drugs** for each test”. The date was tracked and reported at key steps during assay processing. The sensitivity and specificity of each of the three novel tests (MODS, LPA, PSQ) when compared to the reference standard (MGIT) served as an additional measure of effectiveness. The presence or absence of an interpretable result was also recorded.

#### Costs

##### Test-specific materials and personnel costs

The local costs of test-specific laboratory materials and personnel costs were collected at each site using a survey for each diagnostic test using local currency. To ensure accuracy and uniformity of data collection across sites, staff were trained via web- and tele-conferencing on how to complete the surveys. Surveys were completed 3–6 months after enrollment began to allow sites to develop proficiency on any new tests. Costs were listed per batch, and information was gathered on the mean batch size. Site staff were instructed to exclude research-specific costs. When processes were shared across tests, the full cost of that process was counted for each test. Completed surveys were reviewed by investigators at the coordinating site. Missing data or inconsistencies were identified, and sites were asked to clarify the data.

##### Test-specific equipment costs

Although some equipment was already owned by the study sites, we applied the 2010 purchase price for all required equipment and amortized these costs over a recommended useful life of ten years to be consistent across sites [18].

A per sample cost for test-specific equipment was calculated by dividing the total cost by the projected number of samples over ten years using actual test volume. The cost of test-specific equipment per sample was then calculated. Ranges based on volume from the other study sites and a maximum volume was also estimated.

##### General lab equipment and overhead costs

In addition to test-specific materials and equipment, each site had existing general laboratory equipment that was used for the study. Examples are standard beakers, storage cabinets, etc. In addition, there were overhead costs related to building space, utilities, and ongoing maintenance. However, it was not possible to accurately separate general lab equipment and overhead costs used for the current study from those used for non-study clinical services. In addition, general equipment and overhead costs were covered through national government health systems at some sites and were not available. Thus, overhead costs were estimated at 69% of the personnel costs required to deliver the intervention. The overhead cost estimate accounts for facilities costs, indirect support personnel, and other typical indirect costs associated with running a medical clinic [19]. This figure is based on data showing that indirect costs are another 69% above medical personnel costs [20]. This method has been used in other cost-effectiveness analyses [21, 22].

#### Cost analyses

All analyses were conducted from the health care organization perspective. Patient costs were not tracked. All local currency costs were converted to US Dollars using the international currency exchange data reported on XE.com in June 2013. [http://www.xe.com/currencyconverter/] Exchange rates were 1 Dollar = 58.82 Indian Rupees, 12.20 Moldovan Leu, and 9.70 South African Rand. Once converted to US Dollars, personnel costs for India, were $1.52, $1.82, and $4.55/h for an assistant laboratory technician, laboratory technician, and laboratory supervisor, respectively. For Moldova, personnel costs were $1.50, $2.50, or $3.50/h for cleaning personnel, a laboratory technician, and a laboratory supervisor, respectively. For South Africa, all activities were conducted by a laboratory technician at wages of $10.30/h. The mean cost per sample for materials and personnel were calculated separately and then combined in an initial “operations-only” incremental cost-effectiveness analysis. Next, test-specific equipment costs were added to the analysis. A third analysis reflected the addition of overhead costs. Incremental cost-effectiveness ratios (ICER) were calculated for each analysis.

### Sensitivity analyses

Sensitivity analyses were used to explore how the incremental cost-effectiveness analysis results changed when inputs were varied. Inputs that either varied across sites or may vary considerably under other conditions included batch size, hourly wage for laboratory personnel, and lifetime samples processed for test-specific equipment. Thus, it is informative to study the impact of these variables on the study results [23]. Sensitivity analyses were conducted by entering the high and low value from the range of values explained below into the Excel spreadsheets used for calculations. Each cost component (materials, personnel, test-specific equipment, and overhead) was then recalculated and combined into total cost/sample. Each 1-way analysis examined the individual sensitivity of results when batch, hourly wage, and equipment costs were varied separately. Next, the high and low values for two of the three variables were added together. Two-way sensitivity analyses were run for paired variables of batch size/mean hourly wage and batch size/mean equipment cost. The first two-way pair is of interest because two study sites had both low mean hourly wage and higher batch sizes. The second two-way pair is of interest because high volume sites are likely to maximize batch size and also have a reduced per sample equipment cost while the opposite is true of sites with low testing volumes. Finally, a three-way sensitivity analysis was conducted, co-varying the range of values for laboratory batch size, mean hourly wage, and mean equipment cost. The sensitivity tornado chart was produced using Microsoft Excel add-on software.

Batch sizes varied across the sites depending on patient volumes at each site. Batches of PSQ were limited to a maximum of 12 per batch. Batches of the MGIT test were limited to eight samples per well. Each sample was tested with all four tests, so batches remained in the range of 5–12 per batch so that one test was not lagging behind. We varied batch sizes from the minimum to the maximum reported in our study. We also varied personnel costs by the ranges reported in our study.

As described above, hourly wages ranged from $1.50 per hour to $10.30 per hour. However, personnel costs were also affected by time spent on each task, and sites with lower wages tended to use multiple levels of staffing.

Because the actual volume of tests performed with our study equipment may have significantly underestimated total potential volume per machine, we varied the volume from the lowest seen at our study sites, to a maximum of 2000 DST tests per year. We estimated that the PSQ could use its 96 wells to test 12 samples x seven drugs and one control approximately every two days, providing DST on about 2000 samples annually. While more than 2000 samples could be tested with the other diagnostic tools, we used the 2000 tests maximum to standardize the comparison across tests.

## Results

### Operations cost by test

Demographic information for the sample have been published previously [8], and clinical characteristics are available as Additional file 1: Table S1. Operations costs for each test at the three study sites are presented in Table 1. Materials costs varied considerably between sites for the various diagnostic tests. With the exception of the MODS test, India reported higher expenses for consumable laboratory materials. Personnel effort also varied across sites. For the MGIT culture and DST, Moldova spent twice as many hours completing each batch. For all tests, South Africa had the highest cost/sample, mostly because of higher wages as described above. However, South Africa also reported testing the smallest number of samples per batch for most tests, which increased the per sample cost.

**Table 1:** Operations costs for XDR-TB diagnostic tests by study site (in US $ unless otherwise noted)^a^

	MGIT (culture and DST)			LPA (plus and sl)			MODS			PSQ		
	Moldova	India	South Africa	Moldova	India	South Africa	Moldova	India	South Africa	Moldova	India	South Africa
Materials in $	126	274	187	372	525	338	114	42	161	296	353	325
Personnel (in Hours)	8.7	5.7	4.0	12.2	17.0	16.0	6.0	5.5	5.5	11.3	8.5	12.0
Personnel cost $/batch	23	11	41	33	47	165	18	20	57	30	28	124
Total cost$/ batch	149	285	228	405	572	503	132	62	218	326	381	449
# samples/batch	8	8	5	12	12	5	4	6	5	11	12	9
Cost/sample in $	18.56	35.65	45.61	33.73	47.69	100.50	32.88	10.35	43.56	29.64	31.76	49.90

^a^Actual costs were tracked once sites had developed test proficiency (performing a test for at least 3 months)

*DST* Drug susceptibility testing, *LPA* Line-probe assay, *MGIT* Mycobacteria Growth Indicator Tube, *MODS* Microscopic-observation drug-susceptibility assay, *PSQ* Pyrosequencing, *US* United States, *XDR-TB* Extremely drug-resistant tuberculosis

### Test-specific equipment costs

Equipment costs are presented in Table 2. It is noted if equipment was used for more than one diagnostic test. The MGIT and PSQ had large equipment costs because they require expensive machinery while MODS had minimal equipment costs.

**Table 2:** Test-specific equipment costs (US$)

GCDD Equipment	use 1	use 2	MGIT	LPAs	MODS	PSQ
MGIT machine BD	MGIT		47,500			
Inverted microscope	MODS				2888	
Pyromark 96 ID	PSQ					81,086
Pyro Vacuum Workstation	PSQ					5053
Pyro Assay Design software	PSQ					943
Pyro IdentiFire software	PSQ					1734
PCR Workstation	LPA	PSQ		3368		3367
Ultrasonic waterbath	LPA	PSQ		2790		2790
Plate shaker 230 V	PSQ					734
Block Heater Digital-230 V	LPA	PSQ		1323		1323
Water bath - heat	LPA	PSQ		593		593
Uninterruptible power supply	PSQ					544
Twincubator (Hain)	LPA			2441		
Ultrapure water filtration	PSQ					1167
Mini-Plate Spinner Centrifuge	PSQ					1220
Mini Centrifuge	LPA	PSQ		771		771
Mini Vortex	LPA	PSQ		722		722
Totals			$47,500	$12,008	$2888	$102,048
Mean samples/batch (range)			7.0 (5–8)	9.67 (5–12)	5.0 (4–6)	10.67 (9–12)
Samples in equipment lifetime (520 weeks)			3640 (2600–4160)	5028 (2600–6240)	2600 (2080–3120)	5547 (4680–6240)
Mean cost/sample (range by batch size)			$13.05 (11.42–18.27)	$2.39 (1.86–4.47)	$1.11 (0.93–1.39)	$18.40 ($16.35–21.81)
Minimum cost/sample (2000 tests/year)			$2.38	$0.60	$0.14	$5.10

*LPA* Line-probe assay, *MGIT* Mycobacteria Growth Indicator Tube, *MODS* Microscopic-observation drug-susceptibility assay, *PSQ* Pyrosequencing, *US* United States

### Total cost per sample with overhead

Table 3 presents the total costs per sample for each diagnostic test. MODS was cheaper than the MGIT (culture + and DST), with the difference driven by MGIT having a larger equipment cost. The PSQ total costs were slightly higher than MGIT, but considerably less than the LPA tests. The LPA test required little equipment but was more costly due to higher materials and personnel costs associated with conducting both the MTBDRsl and MTBDRplus assays for XDR-TB diagnosis.

**Table 3:** Mean costs /sample adding various components (US$)

Diagnostic Test	Mean Operating Costs	Test-Specific Equipment Costs	Direct Cost Subtotal	General Equipment and Overhead per sample	Total Cost/sample
MGIT	$33.27	$13.05	$46.32	$4.00	$50.32
LPAs	$60.64	$2.39	$63.03	$9.13	$72.16
MODS	$28.93	$1.11	$30.04	$4.35	$34.39
PSQ	$37.10	$18.40	$55.50	$3.90	$59.40

*LPA* Line-probe assay, *MGIT* Mycobacteria Growth Indicator Tube, *MODS* Microscopic-observation drug-susceptibility assay, *PSQ* Pyrosequencing, *US* United States

### Incremental cost-effectiveness

The direct cost subtotal (operations plus equipment costs) was chosen for the final cost-effectiveness analysis because it was more accurately measured than overhead costs which were estimated. Table 4 presents the main incremental cost effectiveness analysis and showed that MODS had a lower cost and a shorter TTR than MGIT, thus dominating that comparison. Ranking third in cost per sample, the PSQ was compared to the previous best choice; MODS. PSQ costs $25.46 more per sample than MODS but was 13.2 days faster, producing an ICER of $25.46/13.2 = $1.93/day saved. Finally, the combined LPA tests were more costly than the previous best choice (PSQ), but did not provide additional effectiveness (time to result) for calculating an ICER.

**Table 4:** Incremental cost-effectiveness of diagnostic tests for XDR-TB

Diagnostic Test	Mean cost /sample ($)	Effectiveness (days to XDR diagnosis)	Incremental cost/sample ($US)	Incremental effectiveness	Incremental cost effectiveness ($/day saved)
MODS	$30.04	14.3 days	–	–	–
MGIT	$46.32	24.7 days	$16.28	dominated	dominated
PSQ	$55.50	1.1 days	$25.46	13.2	$1.93/day saved
LPA plus and sl	$63.03	1.1 days	$7.53	dominated	dominated

*LPA* Line-probe assay, *MDR-TB* Multi-Drug Resistant Tuberculosis, *MGIT* Mycobacteria Growth Indicator Tube, *MODS* Microscopic-observation drug-susceptibility assay, *PSQ* Pyrosequencing, *US* United States, *XDR-TB* Extremely-Drug Resistant Tuberculosis

### Test accuracy

Sensitivity and specificity results for the three rapid tests compared to MGIT DST from sputum for the diagnosis of XDR-TB and MDR-TB are shown in Tables 5 and 6, respectively. Sensitivity and specificity results for the three rapid tests compared to MGIT DST for individual drugs have been previously published [24] but are also available in Additional file 1: Table S2.

**Table 5:** Agreement between three rapid tests and MGIT for detection of XDR-TB

		Sensitivity (95% CI)	Specificity (95% CI)	PPV (95% CI)	NPV (95% CI)	LR+ (95% CI)	LR- (95% CI)	% Agreement (95% CI)
XDR	LPA (*n* = 656)	0.49 (0.36, 0.61)	1.00 (0.99, 1.00)	1.00 (0.87, 1.00)	0.94 (0.92, 0.96)	–	0.51 (0.41, 0.65)	0.95 (0.93, 0.96)
	MODS (*n* = 674)	0.83 (0.72, 0.91)	1.00 (0.99, 1.00)	0.97 (0.88, 0.99)	0.98 (0.97, 0.99)	251 (63, 1003)	0.17 (0.10, 0.28)	0.98 (0.96, 0.99)
	PSQ (*n* = 538)	0.69 (0.56, 0.79)	1.00 (0.98, 1.00)	0.96 (0.85, 0.99)	0.96 (0.93, 0.97)	162 (40, 651)	0.32 (0.22, 0.45)	0.96 (0.94, 0.97)

*CI* Confidence Interval, *LPA* Line-probe assay, *LR-* Negative Likelihood Ratio, *LR+* Positive Likelihood Ratio, *MGIT* Mycobacteria Growth Indicator Tube, *MODS* Microscopic-observation drug-susceptibility assay, *NPV* Negative Predictive Value, *PPV* Positive Predictive Value, *PSQ* Pyrosequencing, *XDR-TB* Extremely-Drug Resistant Tuberculosis

**Table 6:** Agreement between three rapid tests and MGIT for detection of MDR-TB

		Sensitivity (95% CI)	Specificity (95% CI)	PPV (95% CI)	NPV (95% CI)	LR+ (95% CI)	LR- (95% CI)	% Agreement (95% CI)
MDR	LPA (n = 656)	0.99 (0.97, 0.99)	1.00 (0.97, 1.00)	1.00 (0.99, 1.00)	0.97 (0.94, 0.99)	217 (31, 1534)	0.01 (0.01, 0.03)	0.99 (0.98, 1.00)
	MODS (n = 674)	1.00 (0.98, 1.00)	1.00 (0.97, 1.00)	1.00 (0.99, 1.00)	0.99 (0.96, 1.00)	216 (31, 1527)	0.00 (0.00, 0.02)	1.00 (0.99, 1.00)
	PSQ (n = 538)	0.99 (0.97, 0.99)	0.99 (0.95, 1.00)	1.00 (0.98, 1.00)	0.96 (0.91, 0.98)	135 (19, 951)	0.02 (0.01, 0.03)	0.99 (0.97, 0.99)

*CI* Confidence Interval, *LPA* Line-probe assay, *LR-* Negative Likelihood Ratio, *LR+* Positive Likelihood Ratio, *MDR-TB* Multi-Drug Resistant Tuberculosis, *MGIT* Mycobacteria Growth Indicator Tube, *MODS* Microscopic-observation drug-susceptibility assay, *NPV* Negative Predictive Value, *PPV* Positive Predictive Value, *PSQ* Pyrosequencing

### Sensitivity results

**Fig. 1:**
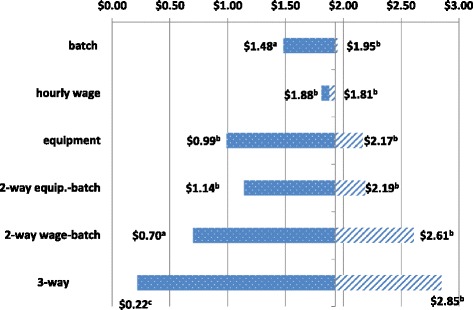
Sensitivity Analyses varying main components of the ICER. **a** LPA ICER ratio relative to the optimal choice of MODS. **b** PSQ ICER ratio relative to the optimal choice of MODS. **c** MODS ICER ratio relative to the optimal choice of MGIT

Analysis inputs (batch size, hourly wage, equipment cost) were varied individually and in combination (Fig. 1). When batch size was maximized using our study site data, LPA became less expensive than PSQ and returns the lowest ICER. In almost all scenarios, MODS remains the least expensive test and is faster than MGIT (Table 5). When hourly wage was varied between $2 and $10 per hour, the ICERs changed very little, ranging from $1.88 to $1.81, respectively. Equipment costs play a significant role in PSQ and MGIT costs, and are affected by equipment lifetime volume. In a low volume site, equipment costs for PSQ increase the ICER to $2.19 per day saved. However, if a site conducted 2000 tests per year over the life of the machines, the ICER for the PSQ compared to MODS is reduced to $0.99.

Figure 1 Sensitivity Analysis Graph of the incremental cost/day saved in diagnosis time by laboratory batch size, mean hourly wage, and mean equipment cost (as a result of testing volume). Solid line reflects decreases, and striped line increases, in the incremental cost-effectiveness ratios as analytic components along the y-axis are varied across the range of values observed in the study.

In two-way sensitivity analyses, varying batch size and equipment from high to low volume provides ICERs of $1.14 to $2.19 for PSQ versus MODS. Varying hourly wage and batch size together produces larger swings in the predicted ICERs. Smaller batch sizes and higher wages ($10) result in an ICER of $2.61 per day saved for PSQ versus MODS. With larger batch sizes and lower wages, the LPA tests again becomes less expensive than PSQ, and provides an ICER of $0.70 per day saved (LPA vs. MODS). Finally, in three-way analysis, small batch size, high wages, and low volume equipment use result in the highest cost per day saved ($2.85). while large batches, low wages, and a high sample volume provide the lowest ICER of $0.22, with MGIT becoming less expensive than MODS.

## Discussion

Using actual study data, MODS is the least expensive test per sample, and is ten days faster than MGIT DST, with good sensitivity/specificity for MDR-TB, but lower accuracy for XDR-TB. The LPA tests (MTBDR*sl* and MTBDR*plus*) and the PSQ provide results in one day, shaving 13 more days off the TTR, but with increased costs. Like MODS, these tests had high sensitivity/specificity for MDR-TB diagnosis, but accuracy drops for XDR-TB.

Despite significantly higher equipment costs per sample, PSQ costs less than LPA overall, especially when volume if high, making it a leading choice for a rapid diagnostic test that can provide a result within one day. Once the PSQ equipment is purchased, the operating costs for this test were about $37/sample in our study, only a few dollars more than MGIT DST with culture and about $9 more per sample than MODS. For clinical sites than cannot afford the PSQ machine, the MODS assay may be a viable and scalable option for detecting XDR-TB in clinical samples. However, MODS may require more intensive biohazard control measures than the molecular assays which do not require growth of TB cultures. However, the difference may be of little impact because most sites would already have safety measures for culture-based testing in place.

When accounting for tests that had to be re-run because of indeterminate or failed tests, we incorporated the proportion of interpretable results for each test by dividing the cost per sample for each test by this ratio, providing a cost per valid result. The PSQ was able to provide a result 84% of the time within 1.1 days, while both the MODS and LPA delivered interpretable results approximately 80% of the time. (Additional file 1: Table S3) Thus, while the rate of indeterminate tests varied by the drug being tested, the PSQ provided significantly more interpretable results overall. This slightly improved the cost per interpretable result for the PSQ relative to the other two tests, potentially enhancing it as a cost-effective choice in some contexts.

To our knowledge, no previous studies have examined the costs of different rapid diagnostic tests for both MDR-TB and XDR-TB, making it hard to interpret our ICER results. However, our cost/sample for the tests are quite similar to those found in other studies. For example, our MGIT DST costs are quite similar to previous figures of about $37 (operations only) [25] and $56 (overhead) [14] found in previous cost studies. The $36 per sample (half of $72) we found was still higher than those found for either LPA test in previous studies ($23–26) [26, 27], primarily because of high costs at our South Africa site (lower volume and batch size with high wages). When omitting South Africa site data, costs become very close to previous results. While previous studies conclude that MODS is a low-cost method for detecting active TB, almost all use only materials costs and do not report a detailed cost analysis [28, 29]. One study estimated the overall costs of using MODS for 1st line drugs and reported costs in Peru around $5 per sample [30]. Our study is the only known study to report a comprehensive cost analysis of MODS for obtaining an XDR diagnosis. Thus, good comparisons were not available for the cost of MODS in our study.

Costs varied considerably across our three study sites. Materials costs were subject to fluctuation because of availability, delivery costs, and currency conversion rates. Personnel costs were about four to five times higher in Port Elizabeth, South Africa than in Moldova or India, partially because they employed a single higher level laboratory technician for the study while the other sites employed multiple levels of staff, including lab assistants. It is notable that the higher paid personnel in South Africa required less time to accomplish most diagnostic tests. Batch sizes also tended to be the smallest in South Africa, due to a lower volume of participants recruited per week, which may be unique to the research environment. The higher costs in South Africa did not result in better test performance on the PSQ [31].

In sensitivity analyses, varying these basic values obtained from our sites, our results were insensitive to most of the assumptions tested, providing a similar result in most cases. A few exceptions worth noting are that, when batch size was maximized by itself or in addition to low hourly wages the LPA became less expensive than PSQ, independent of indeterminate/failed test rates. However, when equipment costs were also minimized through high volume use in addition to max batch size and low hourly wages, the MGIT is the least expensive test and PSQ becomes less expensive than LPA once again. This scenario is not unrealistic for clinical sites in many countries where DR-TB is prevalent and wages are low.

Our study was limited to using TTR and accuracy of the three rapid diagnostic tests as a measure of effectiveness because to properly compare the tests, it was important to conduct all four tests with every study sample. This means treatment decisions and treatment outcomes could not be assigned to a given test. In addition, the sites varied in their familiarity with some tests and thus would vary on which test they used to make treatment decisions. Therefore, we were unable to study the down-stream impact of shorter TTR and/or reductions in sensitivity or specificity on treatment and health outcomes of study patients while comparing the tests. However, a shorter TTR is of obvious importance. In all three of the countries studied, many patient travel significant distances to receive health care from remote locations. With many people traveling 6–12 h or more to receive care, keeping them at the facility or nearby for 1–2 days while DR-TB presence or absence is confirmed may have a major impact on both the spread of the infection as well as on the length and quality of life of the patient that presented.

### Other limitations

As part of our study, sites were not eligible for discounts on equipment or materials for conducting certain tests. These discounts can be sizable for developing countries [14], and should be considered when making decisions on diagnostic tools for detecting DR-TB.

The current study did not quantify and report the amount of time it took to train laboratory personnel on each of the four tests because not all tests were new. Thus sites differed in their familiarity with the tests making it difficult to provide an accurate and comparable summary of training time and experience.

While our inclusion of three different sites is a strength, sites varied considerably in the costs they paid for materials, in their personnel structure, and in wages. Thus, using the mean cost/sample across the three sites may not always provide the best comparison when generalizing the results to other clinical settings. Costs by site are presented, allowing readers to make the most appropriate comparisons for their needs. However, even site specific costs are affected by currency exchange rates and changing availability of materials. The site in South Africa was a high volume site but could only conduct all four research tests on a more limited set of samples. Thus, test costs were likely inflated because of the limited volume of samples studied.

Finally, the accuracy of the rapid tests is based on the assumption that MGIT DST is 100% accurate, which is likely not the case. It is possible that one or more of the rapid tests may be more accurate, which would change the results and conclusions. Further research in this area is needed to determine this.

## Conclusion

In conclusion, our analysis presents the costs associated with three rapid diagnostic tests with good accuracy for the detection of XDR-TB. MODS typically provided a quicker time to result and was less expensive than MGIT DST. The PSQ and LPA tests both provided results much more rapidly and had similar sensitivity and specificity. However, testing volume, the upfront cost of expensive equipment, and potential discounts for developing countries should be considered when deciding which diagnostic test to use.

Our study demonstrates that there are many different factors that affect the actual cost of conducting rapid tests for XDR-TB in clinical practice. Equipment costs, laboratory materials costs, testing volume, and monetary exchange rates are all very important, as are levels of existing laboratory infrastructure. The estimated costs to conduct each test in our study were very similar to those found in previous studies, confirming the relevance of our results. The results allow clinical sites and organizations to roughly estimate their own costs based on characteristics of the three clinical sites in our study. The rapid diagnostic tests studied offer TB clinics and health organizations a variety of options for improving their time to result for XDR diagnosis, depending on their economic options. Our study forms a solid comparator for future cost-effectiveness studies of XDR-TB diagnostic technologies.

## Additional file

Additional file 1: **Table S1** Clinical and Laboratory Characteristics of the Patients. **Table S2** Agreement between three rapid tests and MGIT for detection of resistance for isoniazid (INH), rifampin (RIF), amikacin (AMK), capreomycin (CAP), kanamycin (KAN), moxifloxacin (MOX), and ofloxacin (OFX). **Table S3** Proportion of total assay runs that produced interpretable results from three diagnostic platforms (LPA, PSQ and MODS) with the ability to detect resistance to isoniazid (INH), rifampin (RIF), amikacin (AMK), capreomycin (CAP), kanamycin (KAN), moxifloxacin (MOX), and ofloxacin (OFX). (DOCX 35 kb)

## Abbreviations

DR-TB: Drug-resistant tuberculosis
DST: Drug susceptibility testing
GCDD: Global Consortium for Drug-resistant TB Diagnostics
ICER: Incremental cost-effectiveness ratios
LPA: Line-probe assay
MDR-TB: Multi drug-resistant TB
MGIT: Mycobacteria Growth Indicator Tube
MODS: Microscopic-observation drug-susceptibility assay
PSQ: Pyrosequencing
TB: Tuberculosis (TB)
TTR: Time to result
XDR-TB: Extremely drug-resistant TB
