# Supplementary material for: Cost analysis of rapid diagnostics for drug-resistant tuberculosis
Source: BMC Infect Dis. 2018 Mar 2;18:102. doi: 10.1186/s12879-018-3013-0 (PMC5833048; doi:10.1186/s12879-018-3013-0)
Supplement: Supplementary file 1 — Table S1 Clinical and Laboratory Characteristics of the Patients. Table S2 Agreement between three rapid tests and MGIT for detection of resistance for isoniazid (INH), rifampin (RIF), amikacin (AMK), capreomycin (CAP), kanamycin (KAN), moxifloxacin (MOX), and ofloxacin (OFX). Table S3 Proportion of total assay runs that produced interpretable results from three diagnostic platforms (LPA, PSQ and MODS) with the ability to detect resistance to isoniazid (INH), rifampin (RIF), amikacin (AMK), capreomycin (CAP), kanamycin (KAN), moxifloxacin (MOX), and ofloxacin (OFX). (DOCX 35 kb) [file 12879_2018_3013_MOESM1_ESM.docx]

Additional file 1

Table S1 Clinical and Laboratory Characteristics of the Patients.

| Variable | Value | India | Moldova | South Africa | Total |
| --- | --- | --- | --- | --- | --- |
| Number of patients – n |  | 612 | 254 | 262 | 1128 |
| *Screening Criteria*  a) AFB smear+ within 14 days – n (%) | Yes  No | 612 (100)  - | 254 (100)  - | 262 (100)  - | 1128 (100)  - |
| b) Previously received >1 month of treatment for prior TB episode – n (%) | Yes  No  Unknown | 526 (86.0)  38 (6.2)  48 (7.8) | 59 (23.2)  192 (75.6)  3 (1.2) | 254 (97.0)  8 (3.0)  - | 839 (74.4)  238 (21.1)  51 (4.5) |
| c) Close contact with known drug-resistant TB case – n (%) | Yes  No  Unknown | 169 (27.6)  208 (34.0)  235 (38.4) | 197 (77.5)  51 (20.1)  6 (2.4) | 29 (11.1)  167 (63.7)  66 (25.2) | 395 (35.0)  426 (37.8)  307 (27.2) |
| d) Failing standard TB treatment – n (%) | Yes  No  Unknown | 487 (79.6)  112 (18.3)  13 (2.1) | 16 (6.3)  236 (92.9)  2 (0.8) | 25 (9.5)  234 (89.3)  3 (1.2) | 528 (46.8)  582 (51.6)  18 (1.6) |
| e) Diagnosis of MDR-TB within last 30 days – n (%) | Yes  No  Unknown | 141 (23.0)  398 (65.0)  73 (12.0) | 70 (27.6)  184 (72.4)  - | 26 (9.9)  236 (90.1)  - | 237 (21.0)  818 (72.5)  73 (6.5) |
| BMI – mean, median (range) |  | 17.9, 17.5  (9.0-32.9) | 20.4, 19.9  (12.0-37.2) | 18.4, 17.9  (7.0-32.4) | 18.6, 18.3  (7.0-37.2) |
| HIV Status – n (%) | Positive  Negative  Unavailable | 14 (2.3)  310 (50.6)  288 (47.1) | 2 (0.8)  252 (99.2)  - | 141 (53.8)  105 (40.1)  16 (6.1) | 157 (13.9)  667 (59.1)  304 (27.0) |
| Standardized Results – n (%)  Standardized DST | Smear +  Culture Positive  Pan Susceptible  Mono INH^R^  Mono RIF^R^  MDR-TB  XDR-TB | 511 (83.5%)  492 (80.4%)  75 (15.2%)  19 (3.9%)  2 (0.4%)  335 (68.1%)  54 (11.0%) | 171 (67.3%)  226 (89.0%)  87 (38.2%)  16 (7.0%)  0  102 (44.7%)  14 (6.1%) | 144 (55.0%)  196 (74.8%)  143 (73.3%)  16 (8.2%)  3 (1.5%)  17 (8.7%)  12 (6.2%) | 826 (73.2%)  914 (81.0%)  305 (33.3%)  51 (5.6%)  5 (0.5%)  454 (49.6%)  80 (8.7%) |

Table S2: Agreement between three rapid tests and MGIT for detection of resistance for isoniazid (INH), rifampin (RIF), amikacin (AMK), capreomycin (CAP), kanamycin (KAN), moxifloxacin (MOX), and ofloxacin (OFX).

|  |  | Sensitivity  (95% CI) | Specificity  (95% CI) | PPV  (95% CI) | NPV  (95% CI) | LR+  (95% CI) | LR-  (95% CI) | % Agreement  (95% CI) |
| --- | --- | --- | --- | --- | --- | --- | --- | --- |
| INH | LPA (n=790) | 0.94 (0.91, 0.96) | 1.00 (0.98, 1.00) | 1.00 (0.99, 1.00) | 0.90 (0.86, 0.93) | 264 (37, 1867) | 0.06 (0.04, 0.09) | 0.96 (0.94, 0.97) |
|  | MODS (n=729) | 0.97 (0.95, 0.98) | 0.99 (0.96, 1.00) | 0.99 (0.98, 1.00) | 0.94 (0.90, 0.96) | 74 (24, 228) | 0.03 (0.02, 0.05) | 0.98 (0.96, 0.98) |
|  | PSQ (n=779) | 0.95 (0.93, 0.97) | 0.96 (0.92, 0.98) | 0.98 (0.97, 0.99) | 0.90 (0.85, 0.93) | 24 (13, 46) | 0.05 (0.03, 0.07) | 0.96 (0.94, 0.97) |
| RIF | LPA (n=809) | 0.97 (0.95, 0.98) | 0.98 (0.96, 0.99) | 0.99 (0.97, 0.99) | 0.95 (0.92, 0.97) | 46 (22, 95) | 0.03 (0.02, 0.06) | 0.97 (0.96, 0.98) |
|  | MODS (n=729) | 1.00 (0.98, 1.00) | 0.98 (0.95, 0.99) | 0.99 (0.97, 0.99) | 0.99 (0.97, 1.00) | 45 (20, 99) | 0.00 (0.00, 0.02) | 0.99 (0.98, 0.99) |
|  | PSQ (n=661) | 0.94 (0.91, 0.96) | 0.99 (0.96, 1.00) | 1.00 (0.98, 1.00) | 0.89 (0.84, 0.93) | 104 (26, 414) | 0.06 (0.04, 0.09) | 0.96 (0.94, 0.97) |
| MOX | LPA (n=742) | 0.95 (0.92, 0.98) | 0.99 (0.98, 1.00) | 0.98 (0.95, 0.99) | 0.98 (0.96, 0.99) | 95 (40, 228) | 0.05 (0.03, 0.08) | 0.98 (0.96, 0.99) |
|  | MODS (n=727) | 0.98 (0.95, 0.99) | 0.97 (0.95, 0.98) | 0.95 (0.92, 0.97) | 0.99 (0.97, 0.99) | 34 (20, 58) | 0.02 (0.01, 0.05) | 0.97 (0.96, 0.98) |
|  | PSQ (n=744) | 0.94 (0.90, 0.96) | 0.98 (0.97, 0.99) | 0.97 (0.94, 0.99) | 0.96 (0.94, 0.98) | 55 (28, 110) | 0.06 (0.04, 0.10) | 0.97 (0.95, 0.98) |
| OFX | LPA (n=742) | 0.96 (0.92, 0.98) | 0.99 (0.98, 1.00) | 0.99 (0.96, 1.00) | 0.98 (0.96, 0.99) | 159 (52, 492) | 0.04 (0.02, 0.08) | 0.98 (0.97, 0.99) |
|  | MODS (n=729) | 0.98 (0.96, 0.99) | 0.98 (0.96, 0.99) | 0.97 (0.94, 0.98) | 0.99 (0.97, 1.00) | 49 (25, 93) | 0.02 (0.01, 0.04) | 0.98 (0.97, 0.99) |
|  | PSQ (n=745) | 0.94 (0.91, 0.96) | 0.99 (0.98, 1.00) | 0.98 (0.96, 1.00) | 0.97 (0.95, 0.98) | 111 (42, 294) | 0.06 (0.04, 0.09) | 0.97 (0.96, 0.98) |
| AMK | LPA (n=672) | 0.87 (0.74, 0.94) | 1.00 (0.99, 1.00) | 1.00 (0.90, 1.00) | 0.99 (0.98, 1.00) | - | 0.13 (0.07, 0.26) | 0.99 (0.98, 1.00) |
|  | MODS (n=729) | 0.90 (0.80, 0.96) | 1.00 (0.99, 1.00) | 0.95 (0.86, 0.99) | 0.99 (0.98, 1.00) | 198 (64, 613) | 0.10 (0.05, 0.20) | 0.99 (0.97, 0.99) |
|  | PSQ (n=801) | 0.84 (0.73, 0.91) | 0.99 (0.98, 1.00) | 0.92 (0.82, 0.97) | 0.98 (0.97, 0.99) | 122 (50, 293) | 0.17 (0.10, 0.28) | 0.98 (0.97, 0.99) |
| KAN | LPA (n=672) | 0.48 (0.38, 0.58) | 1.00 (0.99, 1.00) | 1.00 (0.90, 1.00) | 0.92 (0.90, 0.94) | - | 0.52 (0.43, 0.63) | 0.93 (0.90, 0.94) |
|  | MODS (n=729) | 0.62 (0.52, 0.71) | 1.00 (0.99, 1.00) | 0.99 (0.92, 1.00) | 0.93 (0.91, 0.95) | 378 (53, 2693) | 0.38 (0.30, 0.48) | 0.94 (0.92, 0.95) |
|  | PSQ (n=801) | 0.50 (0.41, 0.60) | 0.99 (0.98, 1.00) | 0.92 (0.82, 0.97) | 0.92 (0.90, 0.94) | 69 (28, 167) | 0.50 (0.42, 0.60) | 0.92 (0.90, 0.94) |
| CAP | LPA (n=672) | 0.86 (0.73, 0.94) | 1.00 (0.99, 1.00) | 0.96 (0.84, 0.99) | 0.99 (0.98, 1.00) | 268 (67, 1073) | 0.14 (0.07, 0.27) | 0.99 (0.97, 0.99) |
|  | MODS (n=729) | 0.85 (0.74, 0.92) | 0.99 (0.98, 1.00) | 0.93 (0.83, 0.98) | 0.99 (0.97, 0.99) | 141 (53, 376) | 0.15 (0.08, 0.27) | 0.98 (0.97, 0.99) |
|  | PSQ (n=801) | 0.84 (0.73, 0.92) | 0.99 (0.98, 1.00) | 0.89 (0.79, 0.95) | 0.99 (0.97, 0.99) | 88 (42, 185) | 0.16 (0.09, 0.27) | 0.98 (0.96, 0.99) |

Table S3: Proportion of total assay runs that produced interpretable results from three diagnostic platforms (LPA, PSQ and MODS) with the ability to detect resistance to isoniazid (INH), rifampin (RIF), amikacin (AMK), capreomycin (CAP), kanamycin (KAN), moxifloxacin (MOX), and ofloxacin (OFX).

| N = 914 | **LPA**  (n/%) | | **PSQ**  (n/%) | | **MODS**  (n/%) | |
| --- | --- | --- | --- | --- | --- | --- |
| **INH** | 793/914 | 87% | 786 | 86% | 730 | 80% |
| **RIF** | 812 | 89% | 667 | 73% | 730 | 80% |
| **MOX** | 745 | 82% | 749 | 82% | 730 | 80% |
| **OFX** | 745 | 82% | 749 | 82% | 730 | 80% |
| **AMK** | 675 | 74% | 806 | 88% | 730 | 80% |
| **CAP** | 675 | 74% | 806 | 88% | 730 | 80% |
| **KAN** | 675 | 74% | 806 | 88% | 730 | 80% |
| **mean** | 731.4 | 80% | 767 | 84% | 730 | 80% |
